# Probiotic Supplements: Their Strategies in the Therapeutic and Prophylactic of Human Life-Threatening Diseases

**DOI:** 10.3390/ijms222011290

**Published:** 2021-10-19

**Authors:** Mahmoud Youssef, Hanaa Y. Ahmed, Abel Zongo, Ali Korin, Fuchao Zhan, Essam Hady, Muhammad Umair, Muhammad Shahid Riaz Rajoka, Yongai Xiong, Bin Li

**Affiliations:** 1 College of Food Science and Technology, Huazhong Agricultural University, Wuhan 430070, China; mahmoudyoussef@azhar.edu.eg (M.Y.); patarbtaale@gmail.com (A.Z.); alikorin@azhar.edu.eg (A.K.); lafar@webmail.hzau.edu.cn (F.Z.); essamhady@azhar.edu.eg (E.H.); 2 Food Science and Technology Department, Faculty of Agriculture, Al-Azhar University, Cairo 11651, Egypt; 3 The Regional Center for Mycology and Biotechnology, Al-Azhar University, Cairo 11787, Egypt; hanaa_hyk@yahoo.com; 4 Biological Sciences, Food and Nutrition Research Center, Department of Biochemistry and Microbiology, University Joseph Ki-Zerbo, Ouagadougou 03 BP 7021, Burkina Faso; 5 Department of Food Science and Engineering, College of Chemistry and Engineering, Shenzhen University, Shenzhen 518060, China; umair_uaf@hotmail.com (M.U.); shahidrajoka@yahoo.com (M.S.R.R.); 6 Department of Pharmaceutics, Key Laboratory of Basic Pharmacology of Guizhou Province and School of Pharmacy, Zunyi Medical University, Zunyi 563003, China

**Keywords:** cancer, short-chain fatty acids, COVID-19, chronic diseases, immune system, bacteriocin

## Abstract

Chronic diseases and viral infections have threatened human life over the ages and constitute the main reason for increasing death globally. The rising burden of these diseases extends to negatively affecting the economy and trading globally, as well as daily life, which requires inexpensive, novel, and safe therapeutics. Therefore, scientists have paid close attention to probiotics as safe remedies to combat these morbidities owing to their health benefits and biotherapeutic effects. Probiotics have been broadly adopted as functional foods, nutraceuticals, and food supplements to improve human health and prevent some morbidity. Intriguingly, recent research indicates that probiotics are a promising solution for treating and prophylactic against certain dangerous diseases. Probiotics could also be associated with their essential role in animating the immune system to fight COVID-19 infection. This comprehensive review concentrates on the newest literature on probiotics and their metabolism in treating life-threatening diseases, including immune disorders, pathogens, inflammatory and allergic diseases, cancer, cardiovascular disease, gastrointestinal dysfunctions, and COVID-19 infection. The recent information in this report will particularly furnish a platform for emerging novel probiotics-based therapeutics as cheap and safe, encouraging researchers and stakeholders to develop innovative treatments based on probiotics to prevent and treat chronic and viral diseases.

## 1. Introduction

Chronic diseases, such as cardiovascular, gastrointestinal, and malignant tumors, are the most complicated challenges to health systems overall, representing over 70% of all annual fatalities worldwide [1]. Smoking, an unhealthy diet, exercise inactivity, genetics, environmental agents, and lifestyle are the common risks associated with developing chronic disease. Gut dysbiosis refers to the changes in the quantitative and qualitative composition of microbiota, which can lead to altered host microbial interactions, which can contribute to the development of many chronic diseases, many of which are associated with inflammation [2]. These risks usually coexist and react with each other, leading to an increase in developing chronic diseases.

Furthermore, viral infections represent a significant danger to humanity. Viruses can spread swiftly among humans, leading to global pandemics, especially respiratory viruses belonging to the coronavirus family. In the last two decades, two deadly coronavirus strains have caused a world epidemic outbreak; SARS-CoV-1, discovered in China in 2002, and MERS-CoV, recorded in Saudi Arabia in 2012. Finally, in early 2020, China announced an outbreak of SARS-CoV-2, called COVID-19, associated with a high spread between people and high mortality more than SARS-CoV-1 and MERS-CoV [3]. 

The burden of those threats leads to a significant loss of human lives and negatively influences the socioeconomic status overall, which certainly requires novel and safe therapeutic procedures. Nutraceuticals and functional foods could be an alternative solution for chronic and viral disease treatment. Probiotics are excellent nutraceuticals and functional foods that have significant attention in the food and pharmaceutical sectors. Probiotic-based products captivate consumers with their health benefits and biotherapeutic effects. In 2013, the value of probiotic commerce reached USD 32.06 billion, and it was evaluated to reach over USD 73.8 billion by 2024 [4]. In early 2020, probiotics were also adopted to enhance human immune functions in COVID-19 pandemic treatment guidelines [5]. 

Indeed, specialists have made several attempts to discover probiotics’ role in treating human diseases. However, probiotics’ physiological functions in preventing and treating chronic diseases and viral infections are still poorly understood. Therefore, the current article aims to give insights into probiotics’ critical role against chronic diseases and the influence of probiotics and their assistance in improving immunity and reducing the severe symptoms during COVID-19 infection. The present review is expected to support a better understanding of the physiological functions of probiotics as a prophylactic for chronic diseases, as well as COVID-19, and encourage the development of probiotic-based medicines.

## 2. Probiotics and Their Health Benefits

Probiotics are as ancient as human history; they were discovered in ancient, fermented food. The word probiotic originally belongs to the old Greek language (ρo-βio), which refers to ‘for life’. Most probiotics belong to Gram-positive bacteria (*Lactobacilli* and *Bifidobacteria*). *Bacillus coagulans*, *Streptococcus thermophilus*, a strain of Gram-negative bacteria *Escherichia coli Nissle* 1917, and the yeast *Saccharomyces boulardii* are some additional well-known probiotics [6], though additional species and genera are being evaluated for future use. Probiotics are considered as a part of the gut microbiome that constitutes 1 to 3% of body mass, commensals gut bacteria are also helpful organisms that naturally exist in the gut microbiome and help to keep the host environment healthy [7]. Nonetheless, commensals and probiotics play important roles in digestive and immune health [8], including nutrient and vitamin synthesis, host food product metabolism, intestinal barrier strengthening, pathogenic microbe colonization prevention, anti-inflammatory, and immunoregulation [9,10,11]. Both have therapeutic potential, but we concentrated on the therapeutic benefits of probiotic bacteria in our review.

**Figure 1:**
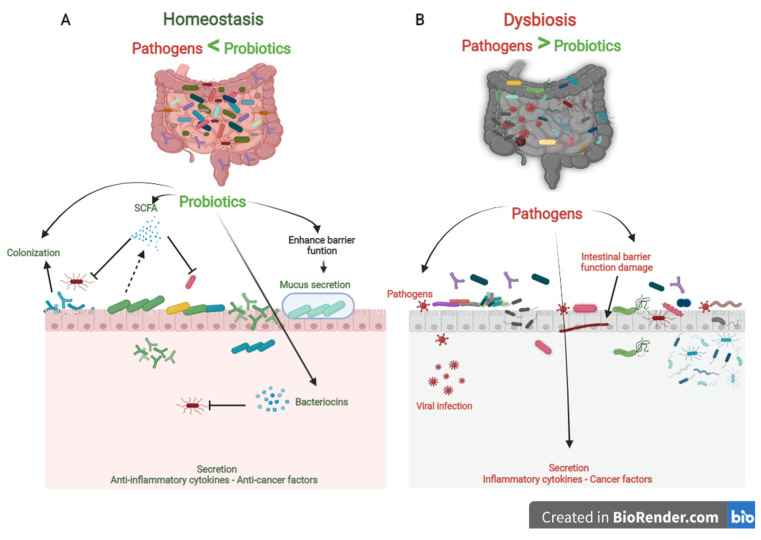
The role of probiotics in competitive exclusion of pathogens. (**A**) Gut microbiota homeostasis refers to probiotics that colonize intestinal epithelial cells. Probiotics produce SCFAs and bacteriocins that prevent viral infection and other pathogens. In addition, probiotics boost anti-inflammatory cytokines and anticancer factors, which prevent the development of chronic diseases. (**B**) In contrast, gut microbiota dysbiosis refers to a decrease in microbial diversity caused by the loss of beneficial bacteria and an increase in pathogen microbiome, which is linked to an increased risk of chronic diseases and viral infections.

Probiotics are the most crucial part of the gut microflora. When administered in sufficient numbers, probiotics colonize different positions in the colon, producing nutrients and energy by fermenting resistant-digestible dietary elements and conferring health advantages to the host, while preserving the homeostasis of the gut microflora [12,13]. As is well known, probiotics are imperative for regulating metabolism, stimulating the immune system against potential infection sources, and preventing chronic diseases [14]. However, factors, such as age, lifestyle, diet, diseases, medicines, and antibiotics, lead to gut dysbiosis. As shown in Figure 1, dysbiosis is the opposite of homeostasis, leading to increased risk factors concerning bacterial and viral infections and chronic diseases [2]. Therefore, maintaining an adequate level of biodiversity is critical for gastrointestinal health.

Probiotics play a vital role in maintaining biodiversity homeostasis in the gut (Figure 1). They compete with pathogens on receptor sites and nutrients in the gut tract, consequently improving gut health and synthesizing different bioactive components, e.g., vitamin B, short-chain fatty acids, bacteriocins [15]. Furthermore, probiotics have their own antiviral, anticarcinogenic, and anti-inflammation effects [16,17]. In addition, probiotics can regulate bowel motion [18], improve cardiovascular functions, and enhance the host’s immune role [19]\. In the following sections, we will briefly debate the role of probiotics as prophylactics in treating chronic diseases and COVID-19 infection (Table A1).

## 3. Probiotics and Competitive Exclusion of Pathogens

The human gut is a complicated ecosystem responsible for a wide range of important biological activities [20]. This ecosystem involves more than 400 anaerobic and aerobic microorganism species, both beneficial and pathogenic, and they are directly affected by the different physiological conditions [21]. The large intestine is considered the final station for this microbiota [22]. Beneficial microbiota and pathogens compete for nutrients, colonize the gut epithelium, and secrete their metabolism products. Probiotics effectively preserve gut microbiota homeostasis by competitive exclusion of pathogenic bacteria. In contrast, if there is a change in the microbial composition that causes an extreme imbalance between the beneficial and potentially pathogenic microorganisms, the gut becomes exposed to colonization of pathogenic with gut microbial changes [23]. So, competitive exclusion refers to a condition in which one species of microorganism competes more strongly than another for receptor sites in the intestinal tract [15,24]. 

Three steps can be mainly described as the competitive mechanisms of probiotics. First, colonization of probiotics into the gut epithelium prevents the fixation of pathogenic bacteria in the gut epithelium. Then, competition for essential nutrients prevents pathogenic microorganisms from obtaining the necessary energy to grow and increase in the gut. Finally, their metabolism products (mucus, bacteriocins, hydrogen peroxide, organic acids, and short-chain fatty acids) may inhibit pathogens (Figure 1). Colonization of probiotics for the mucosal surface area in the human intestinal tract creates a barrier to pathogen growth. Probiotics ably compete with pathogens on epithelial link positions, blocking the intestinal colonization by pathogens, including *Helicobacter*, *Clostridium difficile*, *Clostridium histolyticum*, *Listeria monocytogenes*, *Salmonella Choleraesuis*, *Staphylococcus aureus*, and some strains of *E. coli* and *rotaviruses* [25]. 

Recent studies found a remarkable improvement in the balance of gut microbiota, intestinal cell proliferation, and recovered immune response in children fed with probiotics [26,27]. In addition, probiotic therapies have been shown to reduce the severity of necrotizing enterocolitis, Whipple’s disease, nosocomial and diarrhea, colic, and allergies in several clinical studies [26,28]. The development of those diseases is associated with pathogens.

Probiotics produce a variety of bacteriocins, such as nisin, lactococcin (A, B, Z, G, and Q), pediocin, acidocin, enterocin, enterolysin, and lysostaphin. Bacteriocins are small cationic molecules consisting of 30 to 60 amino acids [29]. These substances have a significant antibiotic and antiviral effect that protects the host from pathogens. Bacteriocins act on the pathogen cytoplasmic layers and target active layer vesicles to damage the proton-motive force and inhibit pathogen replication [15]. Lactococcin (A, B, Z, G, and Q) produced by *Lactococcus lactis subsp.* can increase the permeability of microbial cells by recognizing specific sites in the mannose phosphotransferase system of the responsive cell. Different strains of *Lactococcus lactis subsp. lactis* and *Streptococcus brevis* essentially secrete nisin as abundant as an antibiotic used against a wide range of pathogens [30]. Pediocin, acidocin, enterocin, enterolysin, and lysostaphin released by probiotics powerfully combine with the cell pathogen surface, inhibiting protein synthesis by stopping DNA transcription [29].

On the other hand, short-chain fatty acids (SCFAs) are among the essential postbiotic substances produced during probiotic fermentation of soluble dietary fiber in the human colon. SCFAs are small acid molecules consisting of 2 to 4 carbon atoms in the aliphatic tail, and the most copious in the gut are acetate, propionate, and butyrate. These acids play a pivotal role in gut homeostasis and the competitive exclusion of pathogens. Butyrate in particular, is an essential energy source for the growth and proliferation of epithelial cells. Furthermore, SCFAs have been shown to bestow anti-inflammatory features and immune-strengthening properties and increase antibiotics and antiviral yields [31]. 

## 4. Probiotics and Immune System

**Figure 2:**
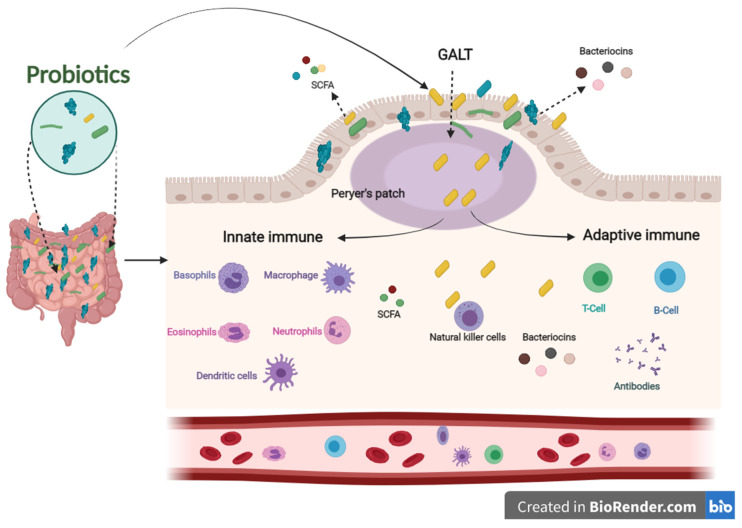
The influence of innate and adaptive immune responses by probiotics. When probiotics travel through Peyer’s patches, lead to an enhanced role of the immune system related to producing antigens and antibodies.

The immune system defends the human body against enemies, such as viruses, bacteria, fungi, foreign matters, and tumor etiology. Generally, the immune system is shaped by two parts: the innate (general) and adaptive (specialized) systems. B and T lymphocytes make up the adaptive immune response, which is related to antigens and antibodies. At the same time, the innate immune system arises when a person is born, which responds to familiar structures called pathogen-associated molecular originals shared by most pathogens [32,33]. The immune system’s integrity is closely related to gut health. A healthier gut confers approximately 25% of the body’s immunity [34]. Gut-associated lymphoid tissues (GALT) make up the most significant part of the innate immune system in the body. GALT consists of functional regions, such as Peyer’s patches, multiple lymphoid follicles surrounded by the mucosa-associated lymphoid tissues (M-cells). Essentially, M-cells are responsible for migrating antigens and microorganisms, including probiotics, from the intestinal lumen to Peyer’s patches through pattern recognition receptors, called Toll-like receptors (TLR). Probiotics, passing through Peyer’s patches, bestow various beneficial impacts on the epithelial layer, contributing to keeping the host’s gut healthier (Figure 2) [35]. 

Concerning the role of probiotics in improving the immune system, several pieces of literature have been published in this regard. Among these studies, Sierra and co-workers have shown that supplementation of *Lactobacillus salivarius CECT5713* in the adult diet enhances immune responses by increasing NK, monocytes, immunoglobulins, and IL-10 cytokines in the plasma [36]. Furthermore, the daily intake of a drink containing *Lactobacillus casei Shirota* improved the expression of the CD69 activation marker T cells and NK cells. In addition, it increased the levels of mucosal salivary IFN-γ, IgA1, and IgA2 in healthy adults [37]. *Lactobacillus gasseri TMC0356* decreases CD28 expression in CD8+ T cells. In contrast, it induces an increase in the number of CD8+ T cells of the elderly [38].

In addition, regular intake of *Bifidobacterium lactis HN019* promotes NK and PMN capacity in the elderly [39]. Two new *Lactobacillus strains* (s193 and s292) isolated from Funazushi (a traditional Japanese fermented food) increase beta-8-integrin on mesenchymal DCs, which are fully activated CD4+ T cells that become Treg cells [40]. 

In addition, postbiotics derived from probiotics contribute to improving the immune system. SCFAs and bacteriocins can directly or indirectly affect the immune system’s capacity against various diseases [41]. SCFAs and bacteriocins are known to stimulate the immune functions of goblet cells. Goblet cells play an important role in barrier restoration by producing mucus and enhancing innate immunity by releasing a variety of influential agents, such as antibiotics, chemokines, and cytokines [42,43]. Those effective agents induce innate immune responses against infections. Similar to the innate immune system, postbiotics have also been confirmed to enhance antibody-specific immunoglobulin and macrophages, dendritic cells, and T cells by boosting the adaptive immune system functions against invaders [44,45]. The results mentioned above indicate that probiotics have an influential role in modulating and stimulating immunological responses to prevent different pathogenic strains, making them a promising therapy for this concern (Figure 2).

## 5. Probiotics and Inflammation

Inflammation is a series of automatic dynamic responses that happen when exposed to infections, external wounds, and toxins in an attempt to self-healing the body [46]. Inflammatory responses include leucocytes, blood serum, and fluids are recruited to the affected site. Generally, the human body releases chemical substances, such as histamine, prostaglandins, leukotrienes, oxygen-and nitrogen-derived free radicals, and serotonin, as part of immune responses to inflammation [47]. Although inflammation is a natural process that defends live cells against damage or pathogens, it can become acute or chronic inflammation. For example, Crohn’s disease, inflammatory bowel diseases, ulcerative colitis, neurodegenerative disorders, allergies, tumors, and heart diseases all cause acute and chronic inflammation [48]. In this regard, more recent attention has focused on using biomedicines, such as probiotics to prevent chronic diseases associated with acute inflammation (Table A1).

Various clinical studies found that consuming probiotic tablets containing *Lactobacillus acidophilus*, *Lactobacillus casei*, *Lactobacillus delrueckii subs. bulgaricus*, *Lactobacillus plantarum*, *Bifidobacterium breve*, *Bifidobacterium longum*, *Bifidobacterium infantis*, and *Streptococcus salivarius subs. Thermophilus* decreased acute inflammation symptoms related to ulcerative colitis and Crohn’s disease [49,50]. Additionally, consumption of *Lactobacillus casei Shirota* significantly reduced the secretion of D3+β7+ integrin cells, IL-4, and CD14+ cells, which are proinflammatory cytokines in healthy adults [37]. *Bifidobacterium breve C50* and *Lactobacillus casei* produce soluble functional elements that remarkably inhibit the NF-κB pathway and TNFα-induced phosphorylation of p38-MAPK in epithelial cells, leading to a decrease in the secretion of pro-inflammatory cytokines [51]. Probiotics reduce the secretion of Th2 cytokines, such as IL-4, IL-5, and IL-13, and decrease IgE levels. On the other hand, probiotic raise C-reactive protein and IgA levels, which help to reduce allergies [52]. In babies with allergies and chronic respiratory diseases, probiotic treatments simulate the cytokine profiles and induce Treg cells parallel with the high concentration of IL-10 and TGF-beta [53].

Many studies have shown the role of postbiotics in suppressing acute inflammation. For example, clinical and animal studies have reported that butyrate and propionate but not acetate inhibit enzymes that mediate inflammation, such as histone deacetylases (HDACs), which play an essential role in gut and lung inflammation and colon cancer [54]. Furthermore, they can also promote IL-18 production [44] and the forkhead box P3 gene with Treg cell differentiation [55], which are recognized to repress gut inflammation and inflammation-related cancers. Bacteriocins can also inhibit the production of interleukin-8 by *Enterococcus faecalis* in gut epithelial cells [56]. Similarly, ancovenin, a cinnamycin-like lantibiotic, inhibits inflammatory responses indirectly by separating phosphatidylethanolamine, which is a term for inactivating phospholipase A2 [57]. Conclusively, clinical experiment outcomes showed the efficient role of probiotics in regulating inflammatory and allergic responses and confirmed that daily consumption of probiotics is helpful in preventing chronic inflammation and allergy.

## 6. Probiotics and Gastrointestinal Dysfunctions

Irritable bowel disease, severe diarrhea, acute constipation, inflammatory gut disorders, and ulcerative colitis are among the gastrointestinal dysfunctions associated with dysbiosis [58]. Halfvarson and co-workers attempted to study the impact of gut microbiota dysbiosis on the development of gastrointestinal dysfunctions. The study was carried out on a gastrointestinal dysfunction group of 137 cases divided into (49 Crohn’s disease, 60 ulcerative colitis, 4 lymphocytic colitis, and 15 collagenous colitis, 9 cases ascontrol). They discovered a significant decrease in the ratio of beneficial gut microbiota and short-chain fatty acids in gastrointestinal dysfunction patients compared to healthy people, highlighting the link between dysbiosis and gastrointestinal dysfunction [18]. Probiotics can drive the gut microbiota towards homeostasis and help relieve gastrointestinal dysfunctions.

Various studies have assessed the efficacy of probiotics in treating gastrointestinal dysfunctions (Table A1). In a recent study, Castell and others observed that *Lactobacillus plantarum L15* lightens ulcerative colitis by repressing LPS-mediated NF-κB activation in-vivo [59]. Likewise, probiotic strains, including *Bacillus spp.* and *Lactobacillus spp.*, have positively alleviated ulcerative colitis by regulating inflammatory agents and protecting the colonic mucosa from injury [60]. In addition, *Lactobacillus fermentum CECT5716*, *Lactobacillus salivarius CECT5713*, *Escherichia coli Nissle 1917*, and *Saccharomyces boulardii CNCMI-745* can also reduce inflammatory cytokines (TNF-α and IL-1β) and increase anti-inflammatory IL-10. They also promote tight junction peptides and restore the colon’s mucosal barrier function [61]. As a result, probiotics may aid in the reduction of cell damage caused by inflammatory bowel disease [62]. In addition, *Bifidobacterium infantis 35624* or *Bifidobacterium lactis CNCM I-2494* combined with other probiotic strains, such as *Streptococcus*, *Lactobacillus*, and *Lactococcus* have been shown to relieve pain in people suffering from irritable bowel syndrome [63,64]. In contrast, using a single strain did not affect pain relief in more than 200 irritable bowel syndrome patients [65].

Clinical trials on 56 adults with functional constipation who received high doses of *Lactobacillus reuteri DSM-17938* for 105 days showed improved gut movement. *Lactobacillus reuteri* alleviates constipation by increasing serum levels of serotonin and brain-derived neurotrophic factor [66]. Brain-derived neurotrophic factors and serotonin are neurotransmitter molecules, playing a vital role in brain-gut communication. These molecules control mobility, secretory function, and neural gut function [67]. Further research on 16 constipated people revealed that taking *Lactobacillus casei strain Shirota* for 28 days significantly increased excretion rates and decreased constipation-related symptoms [68]. Furthermore, recent research found that *Lactobacillus spp.* and *Bifidobacterium spp.* have improved gut movement and ameliorated constipation in Parkinson’s disease patients [69]. Additionally, meta-analysis research on 2327 topics related to the influence of probiotics on chronic constipation in individuals revealed that oral intake of probiotics, particularly multi-strain probiotics, can primarily shorten gut transit time and increase fecal regularity [70]. 

Nonetheless, a clinical study indicated that butyrate treatment significantly reduced white blood cell count, red blood cell sedimentation rate, the inflammatory response of NF-kappaB, and IL-1beta in patients with Crohn’s disease [71]. These findings also suggest the critical role of SCFAs generated by probiotics in treating gastrointestinal dysfunction. As known, gastrointestinal disorders are marked by gut dysbiosis, which significantly reduces SCFAs production. SCFAs, especially butyrate, are responsible for providing energy to intestinal epithelial cells and play a crucial role in restoring intestinal barrier function. In addition, previous studies conducted in rats support the vital role of SCFAs in alleviating intestinal inflammation. SCFAs may initiate signaling cascades that regulate immune response and inflammatory response by signaling through surface G-protein coupled receptors (GPCRs), such as GPR41, GPR43, and GPR109A [72].

## 7. Probiotics and Cardiovascular Diseases

Cardiovascular diseases are the first leading cause of fatality globally. Obesity, hyperlipidemia, diabetes mellitus, lifestyle, and hypertension are risk factors for cardiovascular diseases [73]. It has been confirmed that the gut influences those factors, consequently playing an influential role, in cardiovascular diseases, even though not entirely identified [74,75]. As is known, gut homeostasis maintenance by probiotics reinforces the essential function of organs linked to the cardiovascular and metabolism. In contrast, dysbiosis caused by pathogenic and other external factors results in dysfunction of the host’s organs and tissues, resulting in an elevated risk of cardiovascular diseases [76]. Indeed, the interplay between the intestinal microbiota and the host’s organs and tissues remains an essential factor in understanding the roles of gut microbiota, either probiotics or pathogenic, on cardiovascular diseases (Table A1). 

In a placebo-controlled trial, sixteen patients with atherosclerotic plaque, randomly chosen from a large group, received high doses of *Lactobacillus plantarum (DSM 9843)*. The probiotic treatment effectively helped reduce atherosclerotic plaque by altering gut microbiota and increasing short-chain fatty acids compared to the placebo group [77]. In addition, *Akkermansia muciniphila* effectively inhibited atherosclerotic injury in the Apoe^−/−^ mice by improving TJ proteins’ expression associated with recovering the gut barrier and decreasing the formation of endotoxins-induced inflammation [78]. Besides, probiotics can also regulate the lipid metabolism and decomposition of triethylamine, which block the formation of trimethylamine oxide, a responsible factor for atherosclerosis. 

In addition, a significant decrease in low-density lipoprotein and a boost in high-density lipoprotein have been recorded in patients with hypercholesterolemia after two months of consuming *Lactobacillus acidophilus* and *Bifidobacterium longum* [79]. Furthermore, *Lactobacillus plantarum* has been shown to be helpful in reducing total cholesterol and triglycerides associated with hypercholesterolemia in vivo by increasing high-density lipoprotein and decreasing low-density lipoprotein levels [80]. Thus, high-density lipoprotein plays a vital role in restricting the accumulation of LDL into blood vessel walls and maintaining a low risk of cardiovascular diseases.

On the other hand, hypertension is one of the most important causes of cardiovascular incidents. In this regard, specific probiotic strains, such as *Lactobacillus fermentum*, *Lactobacillus coryniformis*, and *Lactobacillus gasseri*, have shown their ability to prevent hypertension and endothelial dysfunction in rat models [81]. In addition, probiotics have been shown to support endothelial function through increased nitric oxide synthase phosphorylation. Besides, they help restore the balance between T-helper 17 (Th17) and regulatory T-helper (Treg) cells, hence preventing inflammation [81]. Furthermore, recent reports showed a reduced risk of cardiovascular disease in mice fed on probiotics (*Bifidobacterium animalis subsp. lactis F1-3-2* or *Lactobacillus plantarum ZDY04*) combined with a high-choline meal [70,82]. Even further, treatments with *Lactobacillus casei Shirota* and *Lactobacillus gasseri* BNR17 improve obesity and liver damage [83]. Moreover, probiotics may influence fat metabolism and alanine aminotransferase levels in adipose rats [83]. 

Probiotics and their biotherapy properties have shown multiple mechanisms to reduce the risks of cardiovascular disease. First, probiotics help to eliminate cholesterol and prevent the reabsorption of cholesterol in the colon. Second, probiotics accelerate the breakdown of cholesterol through the self-produced BSH-mediated repression of bile acid reabsorption. Third, probiotics modulate gut microbiota and accelerate the discharge of endotoxins and exotoxins into feces. Overall, probiotics have a significant influence in preventing and treating cardiovascular disease. 

## 8. Probiotics and Cancer

Cancer is the second primary reason for fatality globally after cardiovascular diseases. Global cancer cases totaled 18.1 million in 2018, with 9.6 million deaths, and are expected to rise to 29.4 million with 13.2 million fatalities by 2040 [73]. Cancer cells commonly possess characteristics, such as insensibility to standard growth criteria, resistance to apoptosis, limitless proliferation potential, and escape from immune system control. In addition, cancer cells can use different types of nutrients, unlike normal cells; they attack normal cells, unlimited angiogenesis, and metastasis [84]. Cancer is generally generated by a deficiency of DNA repair or mutations during DNA duplication, toxic materials, exposure to hazardous radiation, or according to the genetic history of the individuals [73]. Around 5 to 10% of all cancer cases can be attributed to ancestral genetic mutations. In comparison, 90 to 95% of the cases are linked to other external and internal factors [85].

Gut dysbiosis and the consequent development of pathogenic groups within the gut microbiota can negatively affect either the host’s metabolism or the host’s gut and immune system functionalities, thereby triggering tumor growth [86]. Pathogen microbes are known to be responsible for 20% of tumor growth [87]. Several cancers have been linked to microbial commensal imbalance, or dysbiosis, including esophageal cancer [88], gastrointestinal cancer [89] colorectal cancer [90], pancreatic cancer [91], breast cancer [92], and prostate cancer [93,94]. *Salmonella typhi15* and *Helicobacter spp.16* have been linked to biliary cancer [75,84]. Moreover, *Helicobacter pylori* has been shown to cause gastric cancer [89], among others.

*Shigella flexneri* and *Escherichia coli can* also interfere with DNA damage response and repair pathways, inducing host cell p53 degradation through the secretion of their enzymes inositol phosphate phosphatase D (IpgD) and cysteine protease-like virulence gene A (VirA); therefore, it raises the possibility of starting mutations during the DNA damage response in infected cells [86]. Further, the evidence from in-vivo studies proposes that transplantation of fecal microbes from patients with colorectal cancer can trigger polyp formation, trigger pro-carcinogenic signals, and change the local immune environment in mice compared with healthy controls [95]. Furthermore, evidence from several in vivo models and some clinical studies has linked dysbiosis to colorectal cancer [96].

On the other hand, the use of immune checkpoint inhibitors (ICIs) in cancer treatment, such as monoclonal antibodies targeting the programmed death receptor (PD-1), ligand of programmed death receptor (PD-L1), and cytotoxic T lymphocyte-associated protein 4 (CTLA-4) receptor, is widely used in treatment of many malignancies and is considered a revolution in cancer therapy [97,98,99,100]. The gut microbiota has recently been discovered to significantly influence tumor response to ICIs in both clinical and animal models [101,102,103]. Mouse models were used in some of the early studies on the impact of gut microbiota on the effectiveness of ICIs for a variety of malignancies [104,105]. Tumors from the same mouse strain acquired from various vendors and with diverse gut microbiomes exhibit varied responses to ICIs that target PD-1 for melanoma [104]. Another study was done to see if the link between CTLA-4 monoclonal antibodies and the gut microbiota was the same as in anti-PD1 treatment, and it was observed that *Bacteroidales* play a crucial role in the effects of CTLA-4 inhibition on tumor immunity [105]. Mice with a “beneficial” gut microbiota had a higher response in these trials, which might be due to increased T cell response via antigen presentation cells (APCs) activation. 

The microbiome could be a worthwhile goal to enhance cancer response to treatment. Probiotics have attracted great interest in this concern, owing to their efficient anticancer properties with insignificant or no side effects [24]. Previous studies have suggested that the probiotic can modulate the effectiveness of cancer therapy by modulating metabolism to enhance or suppress the immune response to the tumor or by modulating the metabolism of antitumor factors. Some probiotic strains can modulate the immune response directly. Several in-vitro and in-vivo studies have shown the role of probiotics as anticancer agents (Table A1). For example, Abdolalipour et al. found that injected or oral intake of *Bifidobacterium bifidum* effectually modulated anticancer immune reactions and repressed cancer growth in mice. Intravenous injection, in particular, into cancer-infected mice, activated the antigen-specific IL-12 and IFN-γ, lymphocyte generation, CD8+ cytolytic responses that control and repress tumor spread [106]. Another study showed that administration of *Enterococcus faecalis AG5* (126–168 days, 10^9^ CFU/mL per day) stimulated the production of propanoic acid, which inhibited 5-LOX, enhanced caspase 1p10 production, and induced adipocyte apoptosis in Wistar rats [107]. Moreover, *Lactobacillus plantarum YYC-3* (10^9^ CFU/day) administration for 49 days inhibited the incidence of colorectal cancer and mucosal injury in APC^Min/+^ mice fed fatty meals [108]. Other studies indicated the role of probiotics in inhibiting colorectal cancer in the rat model. This research demonstrated the protective effects of *Lactobacillus lactis subsp. lactis (R7)*, *Lactobacillus fermentum*, *Lactobacillus plantarum*, and *Lactobacillus salivarius Ren* on the development of colorectal cancer in rats [109,110,111]. Several studies have shown that specific probiotic strains inhibit pathogens typically present in the gut, including but not limited to pathogenic variants within the *Escherichia coli species*, *Salmonella enterica*, and *Clostridium perfringens* [112,113]. These pathogens have been associated with the secretion of enzymes, such as beta-glucuronidase, azoreductase, and nitroreductase. These enzymes can transform procarcinogens into carcinogens [114,115]. Finally, the role of probiotics as anticancer and anti-mutagenic agents can be summarized as follows:

- Maintenance of gastrointestinal homeostasis.
- Transformation and lysis of mutagens and carcinogens present in the gastrointestinal tract.
- Secretion of specific postbiotics with anticarcinogenic action, for instance, short-chain fatty acids and bacteriocins.
- Enhancing metabolism and increasing nutrient absorption.
- Accelerates apoptosis and supports DNA repair.
- Simulation of the immune system’s roles.

## 9. Probiotics and COVID-19 Infection

Over 300 viruses have been discovered to infect human beings worldwide, and the total number of viruses is gradually increasing each year [116]. Recently, in late 2019, a novel coronavirus (SARS-CoV-2) emerged as a dangerous threat to all humankind. Even though all ages are susceptible to contracting COVID-19, people with chronic diseases develop acute symptoms more than healthier people. Despite the emergency approval of a few COVID-19 vaccines, the infection and fatality rates are still rising. As of 2 June 2021, over 170 million cases worldwide were recorded, with more than 3.5 million deaths [117]. Therefore, other sources of therapeutics could help address the dire consequences of COVID-19 and its variants. 

**Figure 3:**
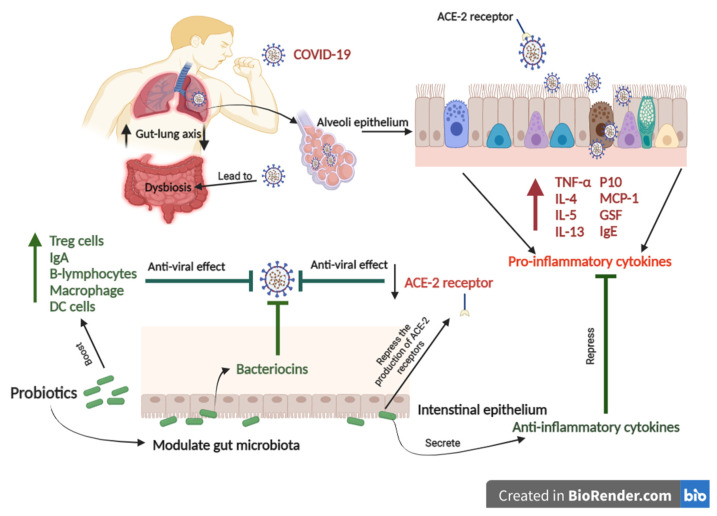
The schematic figure shows the potential mechanism of probiotics to combat COVID-19. COVID-19 binds to the ACE2 receptor that allows the virus to infect the lung alveoli epithelium. COVID-19 leads to gut-lung microbiota dysbiosis associated with increased levels of pro-inflammatory cytokines. In contrast, Probiotics can modulate gut-lung microbiota and repress the production of ACE-2 receptors. Probiotics can also enhance immune responses to prevent COVID-19.

COVID-19 infects the oropharyngeal epithelial cells, causing lung tissue deterioration and hyper inflammation, resulting in acute respiratory distress (Figure 3) [118]. Coronavirus entry is mediated by the viral spike (S) glycoprotein. Then, COVID-19 is linked to the angiotensin-converting enzyme 2 (ACE2) [119]. The virus’s association with ACE2 allows it to attack cells in the oropharyngeal epithelium, resulting in lung injury and hyper-inflammation [120,121]. The angiotensin-converting enzyme 2 (ACE2) is highly expressed in gut and lung tissues, contributing to well-described symptoms in COVID-19 infection [122]. The most severe cases of COVID- 19 often include pneumonia followed by acute respiratory syndrome [123]. These cases also involve hypoxemic respiratory distress concurrent with lung neutrophilia, mucus and fluid accumulation in the bronchi, and bronchiectasis [124]. COVID-19 also relates to gastrointestinal syndrome [125]. A clinical trial on 651 patients with COVID-19 in Zhejiang, China, from 17 January 2020 to 8 February 2020, found that 74 patients had at least one gastrointestinal syndrome, including nausea, vomiting, or diarrhea [125]. Acute lung dysfunctions, high lactate dehydrogenase/glucose levels, gut microbiota imbalance, reduced *Lactobacilli* and *Bifidobacteria* were observed in COVID-19 cases with gastrointestinal symptoms [126]. Both gut and lung tissues share a relationship influencing in-inflammatory and immune responses via the gut–lung axis, so that an abnormal function in either of them will cause the installation of the disease in the other [122].

Focusing on gut flora, microbe interactions and their products influence innate and adaptive immune signals and cells locally, systemically, particularly in the lung. It has been shown that the gut microbiome affects susceptibility to asthma, lung allergic responses, and chronic obstructive pulmonary disease [41,127]. Probiotics and their mechanisms of action in the prevention and treating respiratory diseases could be beneficial in the COVID-19 pandemic [128].

In a clinical trial on seventy patients, twenty-eight cases received multi-probiotic strains combined with hydroxychloroquine, antibiotics, and tocilizumab, and forty-two cases received conventional treatment without probiotics [129]. Results showed that patients who received multi-probiotic strains had cured diarrhea and other mild symptoms better than the conventional treatment group; furthermore, the predicted risk of increasing respiratory failure was eight times lower in the probiotic supplemented group.

A few studies reveal that SARS-CoV-2 also impairs immune responses by reducing anti-α-Gal IgE and IgM antibodies, which are considered the first line of defense against viral and bacterial infection. In contrast, it maximizes cytokine levels that cause acute inflammation and the anti-SARS-CoV-2 (Spike) IgG antibody associated with COVID-19 severity [130]. Moreover, a recent study discovered that probiotic supplementation modulates gut microbiota, increasing α-Gal and improving immune response against COVID-19 [130].

In addition, respiratory infections activate Th17 cells, marked by the secretion of interleukin-17 (IL-17) [131]. IL-17 is a highly inflammatory cytokine that moves to the gut through the gut–lung axis, causing inflammations, dysbiosis, and an impaired immune system [2]. Postbiotics produced from probiotics, such as short-chain fatty acids and bacteriocins, have been shown to induce anti-inflammatory T helper (Th) 1, Th17 effector cells, and IL-10 + Tregs, which have been associated with the prevention of respiratory inflammation diseases. In addition, those postbiotics can induce immune responses against respiratory infections through histone deacetylase repression [57]. Furthermore, animal studies have reported that probiotics play a protective role in combatting bacterial and viral pneumonia by boosting innate and adaptive immune responses and suppressing inflammatory responses [122]. Accordingly, the gastrointestinal and respiratory are mutually joined organs through the gut–lung axis, which interacts with the immune system to maintain healthy homeostasis [132]. Achieving gut homeostasis by consuming probiotics is essential for boosting immune responses and repressing inflammation, making probiotics a potentially curative procedure for COVID-19, or at least lessening its acute symptoms and saving lives (Table A1).

## 10. Conclusions

In summary, probiotics are an indispensable part of the gut microbiota and bestow diverse therapeutic and prophylactic attributes when administered in tolerable amounts. Throughout this review, we have highlighted recent evidence in the literature showing the direct and indirect significance of probiotics in preventing and treating diseases. In general, the beneficial attributes of probiotics involve colonization and maintenance of biodiversity homeostasis in the human gut by inhibiting pathogen and secretion of bacteriocins and SCFAs. Furthermore, probiotics modulate pro-inflammatory factor production. In addition, probiotics transform and digest carcinogens. These beneficial attributes play an essential role in preventing and treating specific kinds of chronic diseases. Similarly, probiotics and their postbiotics could provide cardiovascular benefits according to their capacity to reduce hypercholesterolemia, hypertension, and oxidative stress. Besides, probiotics and their metabolites boost the host’s innate and adaptive immune systems.

Furthermore, postbiotics derived from probiotics can interact with the lung-gut axis and play a critical role in combatting COVID-19 and other viral infections. Finally, we are looking through probiotics as part of microbiota populations that live in an interrelated relationship with the host, where probiotics feed on non-digestible macronutrients in the gut to grow and proliferate. In exchange, they confer green postbiotics, such as essential nutrients, vitamins, short-chain fatty acids, and bacteriocins. Those postbiotics have potent therapeutic and prophylactic properties that should motivate researchers, investors, stakeholders, and consumers to invent new medicines based on probiotics to prevent and treat chronic diseases and viral infections.

## Data Availability

Data are available on reasonable request from the authors.

## Appendix A

**Table A1:** Models of human and animal studies regarding the effect of probiotics on human health.

Probiotic Strain	Model of Study	N. Cases	Dosage and Follow-Up	Outcomes	Refs.
**Cardiovascular diseases**					
*B. breve CECT7263 (BFM)* *L. fermentum CECT5716 (LC40)*	Rats	60 males	1 × 10^9^ CFU/daily for 91 days	Increase SCFA production Regulated blood pressure Improve gut microbiota Decreased NADPH oxidase Reproduce the Th17/Treg	[133]
*L. plantarum 299v*	Human	15 men; Ages 40–75	20 × 10^9^ CFU/daily for 42 days	Recovered vascular endothelial function Increase anti-inflammatory response Promoting innate immunity. Suppress inflammatory response. Regulated blood pressure	[134]
*L. johnsonni 3121* *L. rhamnosus 86*	Mice	30 males	1 × 10^10^ CFU/daily for 84 days	Improved lipid metabolism. Suppressed of produce adipocyte. Increase HDL.	[135]
*B. longum BB536 + Red yeast rice*	Human	16 males and 17 females; Ages 18–70	1 × 10^9^ CFU/daily for 84 days	lessened TC, LDL, apoB, and Lathosterol: TC ratio	[136]
*Lactobacilli, Bifidobacteria*, *and Streptococcus thermophilus*	Human	110 subjects; Ages 50–75	9 × 10^11^ CFU/daily for 56 days	Reduced blood pressure Enhanced immune system and anti-inflammation factors	[137]
*B. animalis subsp. lactis CECT 8145*	Human	43 men and 83 women	10^10^ CFU/daily for 90 days	Decreased body mass Improved lipid metabolism	[138]
**Cancer**					
*B. longum*, *Bifidobacterium bifidum*, *L. acidophilus*, *L. plantarum,*	Mice	12 females	30 × 10^9^ CFU per day for 21 days	Decreased the tumor volume Rise apoptotic cells and infiltration of immune cells	[139]
*L.s acidophilus CICC 6074*	Mice	32 females	1 × 10^10^ CFU per day for 28 days	Caused colon cancer apoptosis	[140]
*L. reuteri GMNL-89* *and L. paracaseiGMNL-133*	Mice	--	2 × 10^7^ CFU per day for 28 days	Decreased the pancreas cancer cell proliferation suppress PanIN progression and cancer cell metastasis	[91]
*L. casei TD-2*	Mice-Murine			Inhibited tumor growth. Boosting immune response (IFN–γ, IL-4, and IL-12) Growth apoptotic cells	[106]
**Irritable bowel syndrome**					
*B. coagulans LBSC*	Human	40 cases; Ages 18–65	6 × 10^9^ CFU/daily for 80 days	Enhanced stool consistency Lighten bloating, abdominal pain, diarrhea, constipation, stomach rumbling, nausea, vomiting, headache, and anxiety	[141]
*B. animalis subsp. lactis MN-Gup*	Mice	40 cases	14 days	Increased short-chain fatty acid Recovered functional constipation symptoms Boosting of a balanced composition of microbiota in feces	[142]
	Human	50 adults	28 days		
*B. longum, L. acidophilus*, *L. fermentum, L. helveticus*, *L. paracasei, L. rhamnosus*, *and S. thermophiles*	Human	55 adults (men and women)	1 × 10^10^ CFU/daily for 21 days	Ameliorated gastrointestinal function Alteration of gut microbiota components	[143]
**Stimulate immune response**					
*L. acidophilus LLA-10*, *L. helveticus LLH-108*, *L. rhamnosus LLR-L1*, *L. fermentum LLF-01*, *and L. bulgaricus LLB-06*	Human	40 adults	3 × 10^8^ CFU/twice daily for 21 days	An improvement in IL-10, IL-17A levels, and HLA-DR+ natural killer (NK) cells A notable drop in cytokine IL-6, IFN-γ, TNF-α, and secretory IgA rates	[144]
*L. plantarum Inducia*	Mice	20 mice	2 × 10^8^ CFU/daily for 30 days	The number of ileal and colonic lymphocytes increased Biodiversity in the gut improved	[145]
	Human	12 healthy adults	3 × 10^9^ CFU/daily for 21 days	Manipulating of innate immune (blood monocytes and IL-6)	
*L. rhamnosus GG*	Rats	37 females 23 males	1 × 10^9^ CFU/daily for 20 days	Lessening the 5-HTRs expression Boosting SERT Increase anti-inflammatory response	[60]
**Respiratory virus**					
*S. thermophilus DSM 32345*, *L. acidophilus DSM 32241*, *L. helveticus DSM 32242*, *L. paracasei DSM 32243*, *L. plantarum DSM 32244*, *L. brevis DSM 27961*, *B. lactis DSM 32246*, *B. lactis DSM 32247.*	Human	70 cases; Ages 50–70	Hydroxychloroquine, antibiotics, and tocilizumab, alone or in combination 24 × 10^11^ CFU/daily	Decreased risk of developing respiratory failure Mitigation of diarrhea and other gut symptoms	[129]
*B. infantis*, *L. acidophilus*, *E. faecalis* *and B. cereus*	Human	120 Kids with recurrent respiratory tract infection	For 60 days	Lessened cough, fever, and use of antibiotics	[146]
*L. rhamnosus GG*	Human	209 elderly people	1 × 10^10^ CFU/daily for 180 days	Somewhat decreased the risk of respiratory virus infection	[147]
